# Community-Centered Responses to Ebola in Urban Liberia: The View from Below

**DOI:** 10.1371/journal.pntd.0003706

**Published:** 2015-04-09

**Authors:** Sharon Alane Abramowitz, Kristen E. McLean, Sarah Lindley McKune, Kevin Louis Bardosh, Mosoka Fallah, Josephine Monger, Kodjo Tehoungue, Patricia A. Omidian

**Affiliations:** 1 Department of Anthropology & Center for African Studies, University of Florida, Gainesville, Florida, United States of America; 2 Department of Anthropology, Yale University, New Haven, Connecticut, United States of America; 3 Department of Epidemiology & College of Public Health and Health Professions, University of Florida, Gainesville, Florida, United States of America; 4 Centre for Infectious Disease, The University of Edinburgh, Edinburgh, United Kingdom; 5 Emerging Pathogens Institute, Gainesville, Florida, United States of America; 6 Ebola Emergency-Response Program, Action Contre la Faim (ACF), Monrovia, Liberia; 7 Focusing International, Chestnut Ridge, New York, United States of America; Tulane School of Public Health and Tropical Medicine, UNITED STATES

## Abstract

**Background:**

The West African Ebola epidemic has demonstrated that the existing range of medical and epidemiological responses to emerging disease outbreaks is insufficient, especially in post-conflict contexts with exceedingly poor healthcare infrastructures. In this context, community-based responses have proven vital for containing Ebola virus disease (EVD) and shifting the epidemic curve. Despite a surge in interest in local innovations that effectively contained the epidemic, the mechanisms for community-based response remain unclear. This study provides baseline information on community-based epidemic control priorities and identifies innovative local strategies for containing EVD in Liberia.

**Methodology/Principal Findings:**

This study was conducted in September 2014 in 15 communities in Monrovia and Montserrado County, Liberia – one of the epicenters of the Ebola outbreak. Findings from 15 focus group discussions with 386 community leaders identified strategies being undertaken and recommendations for what a community-based response to Ebola should look like under then-existing conditions. Data were collected on the following topics: prevention, surveillance, care-giving, community-based treatment and support, networks and hotlines, response teams, Ebola treatment units (ETUs) and hospitals, the management of corpses, quarantine and isolation, orphans, memorialization, and the need for community-based training and education. Findings have been presented as community-based strategies and recommendations for (1) prevention, (2) treatment and response, and (3) community sequelae and recovery. Several models for community-based management of the current Ebola outbreak were proposed. Additional findings indicate positive attitudes towards early Ebola survivors, and the need for community-based psychosocial support.

**Conclusions/Significance:**

Local communities’ strategies and recommendations give insight into how urban Liberian communities contained the EVD outbreak while navigating the systemic failures of the initial state and international response. Communities in urban Liberia adapted to the epidemic using multiple coping strategies. In the absence of health, infrastructural and material supports, local people engaged in self-reliance in order to contain the epidemic at the micro-social level. These innovations were regarded as necessary, but as less desirable than a well-supported health-systems based response; and were seen as involving considerable individual, social, and public health costs, including heightened vulnerability to infection.

## Author Summary

In this study the authors analyzed data from the 2014 Ebola outbreak in Monrovia and Montserrado County, Liberia. The data were collected for the purposes of program design and evaluation by the World Health Organization (WHO) and the Government of Liberia (GOL), in order to identify: (1) local knowledge about EVD, (2) local responses to the outbreak, and (3) community-based innovations to contain the virus. At the time of data collection, the international Ebola response had little insight into how much local Liberian communities knew about Ebola, and how communities managed the epidemic when they could not get access to care due to widespread hospital and clinic closures. Methods included 15 focus group discussions with community leaders from areas with active Ebola cases. Participants were asked about best practices and what they were currently doing to manage EVD in their respective communities, with the goal of developing conceptual models of local responses informed by local narratives. Findings reveal that communities responded to the outbreak in numerous ways that both supported and discouraged formal efforts to contain the spread of the disease. This research will inform global health policy for both this, and future, epidemic and pandemic responses.

## Introduction

The West African Ebola epidemic emerged in the forest region of Guinea in late December 2013 and appeared to be contained until May 2014, when it rapidly accelerated its rate of incidence and crossed into urban areas in Sierra Leone and Liberia [1]. Upon entering Sierra Leone and Liberia, the rate of Ebola virus disease (EVD) transmission increased rapidly resulting in 1,711 cases by August 8, 2014, when the WHO declared that the conditions for a Public Health Emergency of International Concern (PHEIC) had been met—the third announcement of its kind in history. Although the tide has since turned, as of January 2015 there were over 21,000 confirmed, probable, or suspected cases of EVD, and more than 8,600 Ebola-related deaths, 3605 of which occurred in Liberia alone [2].

The West African Ebola epidemic has demonstrated that the existing range of medical and epidemiological responses to emergency epidemics is insufficient in some particularly vulnerable post-conflict low-income countries like Liberia and Sierra Leone, while other low-income countries like Senegal and Nigeria are able to rapidly contain outbreaks with international support. This fact has provoked key discussions regarding the need to strengthen African health systems, redress exceedingly poor public health and healthcare infrastructures [3], and examine the capability of local communities to respond to global health crises.

A key lesson from the West African Ebola epidemic is that local community engagement is crucial for response, and may have played a role in the decline in transmission rates [4–7]. In a context in which health surveillance systems had failed, healthcare workers were experiencing disproportionately high mortality rates due to Ebola infection, clinics and hospitals across Liberia were shut down, and the construction of hospitals and Ebola Treatments Units (ETU’s) could not keep pace with demand [8], communities were compelled to generate solutions of their own. The social context for local innovations and response did not appear to be auspicious, as local communities exhibited resistance towards hospitals, ETUs, and mandatory cremation policies [9], and global health experts expressed concern regarding local communities’ abilities to adequately contain the highly infectious disease and treat the sick.

Declining transmission rates are likely to be attributed to multiple factors. Mathematical modeling, having featured prominently in the Ebola response, has been used to understand transmission dynamics as well as the significance of different control interventions [8, 10]. For example, a recent agent-based model found that Ebola treatment centers (ETCs), safe burials, and protection kits played a defining role in case reductions in Liberia [11]. However models are invariably simplifications of complex realities. In a recent commentary, Chowell and Viboud [12] descripted the tendency to simplify mobility patterns, social behavior, and differences between urban and rural settings. Others have discussed how psychological and socio-cultural factors remain unaccounted for in most Ebola transmission and control models [13]. Local mitigation strategies undertaken by communities themselves often remain unaccounted for in causal explanations.

Modeling the effectiveness of specific Ebola-related interventions requires attention to local community and household-based grassroots efforts. Epidemiologists are increasingly recognizing the role of local social networks in controlling infectious disease outbreaks outside of formal public health and biomedical interventions, and they are attempting to account for them in complex models [14–15]. Reports from news outlets, however, anticipated this response, suggesting well in advance of epidemiologists that declining rates of Ebola were due, in large part, to local acceptance of safe burials and the mobilization of communities to isolate and refer infected individuals to Ebola response teams, ETUs, and community-care centers (CCCs). For example, one case-finder in West Point, Monrovia’s largest slum, stated “The virus is in the community, and the best way to take it from the community is for the community itself to take charge” [16]. Joel Montgomery, a CDC team leader in Liberia also noticed that, “Communities are doing things on their own, with or without our support” [17].

Using data collection methods informed by participatory rural appraisal models [18–20], this study shows how community-based responses both supported and discouraged formal efforts to manage the epidemic; and how community-based informal responses may have contributed to containing the outbreak. Specifically, we provide insight into community leaders’ tactics and strategies for managing the presence of Ebola in their communities, and we analyzed these tactics and strategies in order to develop conceptual models of local responses informed by local narratives and descriptions. When reviewed using an inductive approach informed by ethnographic contextualization (see methods section below), the data revealed how communities were engaging in self-reliance—in the absence of health, infrastructural, and material supports—in order to contain the epidemic at the micro-social level.

Community leaders described how they proposed to engage in prevention efforts through training and awareness, hygiene, surveillance, and the creation of local infrastructures; how they proposed to conduct treatment and response through a process of isolation, quarantine, and triage; and how they proposed to manage the sequelae of the presence of Ebola, especially among orphans, survivors (people who had been ill but had recovered), and for memorialization. They also identified critical epidemic-related and long-term structural barriers inhibiting the utilization of public health and medical infrastructures. Importantly, these data are analyzed in this article to present how local caregiving efforts served as both pathways for disease containment and as socially-mediated pathways for potential new infections.

Our findings demonstrate how communities showed resilience, innovation, and rapid response to the Ebola crisis. They also show how under conditions of extreme stress, culture can be flexible and supple in response to extreme circumstances and the arrival of new information (like public health messages), and make allowances in extraordinary conditions [21]. It contributes to a small but growing literature on local understandings and responses to hemorrhagic fever outbreaks [22–26] that challenges conventional thinking about the role that “culture” plays in epidemics. As Hewlett has demonstrated in Ebola outbreaks in the Democratic Republic of the Congo, Uganda, and Gabon [22, 26], and this research demonstrates for Liberia, culture is not a fixed entity. Local knowledge can shift rapidly in response to public health information and local epidemiological realities [27]. Funerary and caregiving practices can be suspended or altered [28]. Families will break with convention to protect uninfected individuals.

The attributes of culture that public health experts often attend to in the Ebola outbreak (like hygiene practices, food practices, and death rituals) may not been the most important factors in the cultural principles that shaped community-centered Ebola responses. Attention needs to shift to *the culture of caregiving* that exists in Ebola-affected cities and towns. We need to better understand how strong and dense the emotional ties that bind families and communities together are and can be, and precisely how Ebola, and the failed Ebola response, is doing violence to those social ties [29–33].

The reports of community leaders presented below are based upon hypothetical questions and conditional inquiries about *best practices for containing Ebola*, and *what they would do if Ebola emerged in their communities*. The community leaders’ responses, however, emerged directly from their lives and plans as they attempted to prevent or contain the epidemics in and around their own Monrovia communities and broader social networks. Therefore, the data reported here are indicative of transient emergent plans then in circulation in Monrovia communities during the height of the outbreak.

## Materials and Methods

These data were collected as part of a Government of Liberia/World Health Organization GOL/WHO rapid assessment of community leaders’ perceptions of appropriate management practices for addressing the incidence of Ebola in their communities. The research teams were trained and directed by an applied medical anthropologist and conducted data collection from September 1-20th, 2014 in 15 communities of varying economic, ethnic, and population characteristics in Monrovia and Montserrado County, Liberia. Data are drawn from focus groups, qualitative field notes, and supporting literatures. Liberian research teams conducted 15 focus groups, one in each community, consisting of 15–20 people of mixed gender, for a total of 368 participants. All participants were community leaders, drawn from women’s groups, youth groups, local zonal heads, political groups, clinics, church-based organizations, non-governmental organizations, and recreational clubs. The tone of the meetings was widely reported as cooperative and participants were positively engaged.

**Fig 1:**
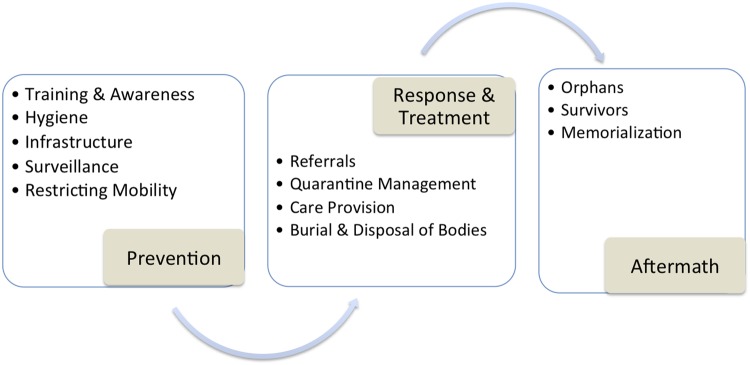
Monrovia community leaders’ representation of optimal community-based care, August—September 2014.

**Fig 2:**
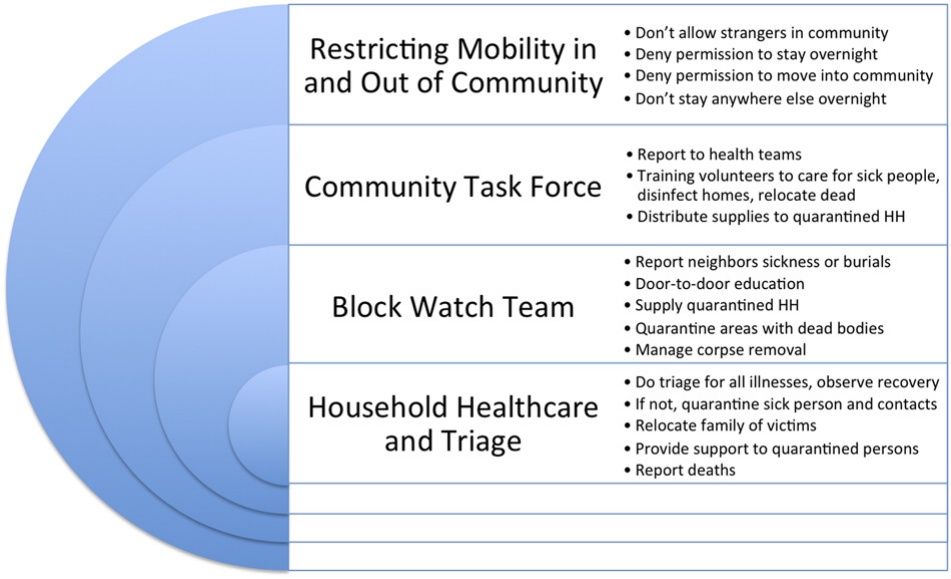
Monrovia community leaders’ detail of optimal community-based Ebola surveillance, August—September 2014.

**Fig 3:**
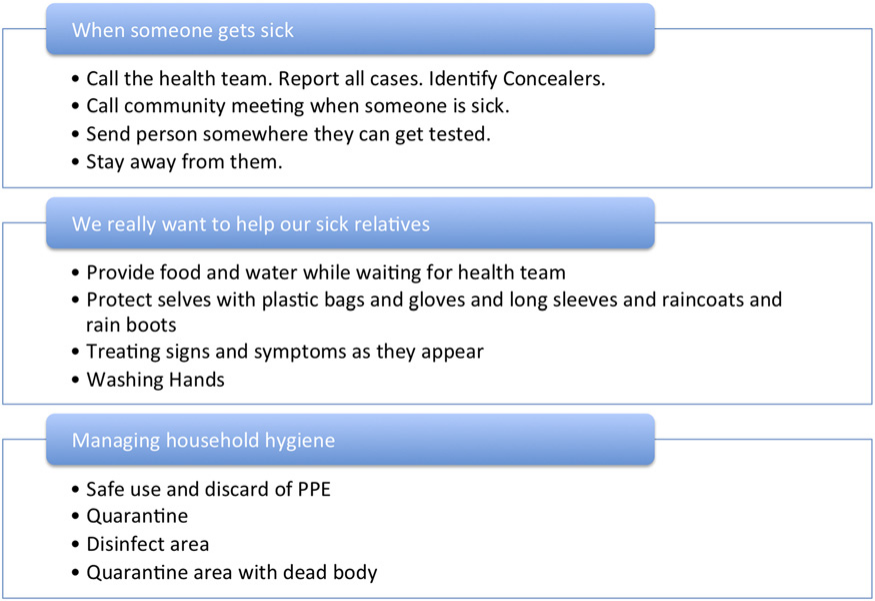
Monrovia community leaders’ narratives describing how to triage sickness in household context, August—September 2014.

**Fig 4:**
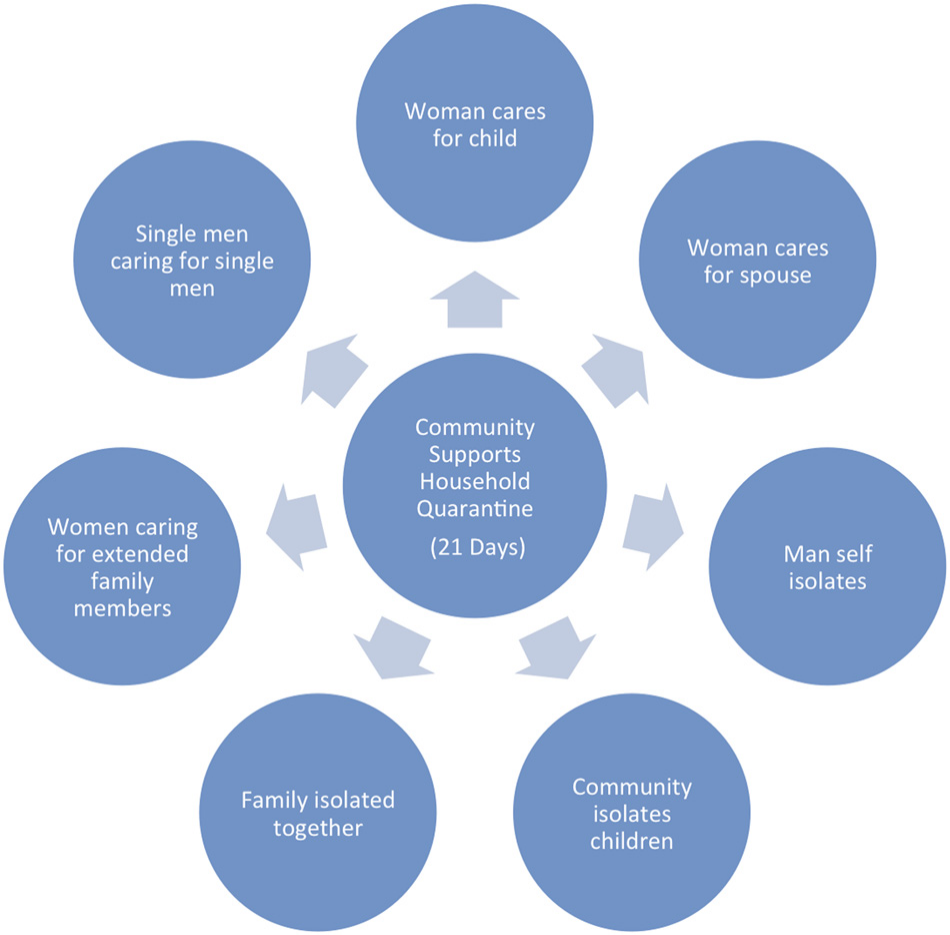
Gendered configuration of Ebola-related caregiving in community-based household quarantine scenarios, August—September 2014.

Field-level ethnographic contextualization was provided through the fieldnotes and qualitative observations of the lead expatriate anthropologist directing the eight-person Liberian research team and the eight Liberian researchers themselves. Field-level observations were based on community-based participant observation, informal interviews, and discussions with various agencies in the Ebola response, as well as focus group data. An additional layer of ethnographic contextualization was provided by the lead author, who has nine years of ethnographic field experience in Monrovia, Liberia [34]. A team of public health and anthropological researchers at the University of Florida and at Yale University analyzed, coded, and thematically clustered de-identified focus group data in October 2014. The inductively derived social structural models (see Figs 1–4) emerge from focus group findings and were informed by the broader literature on the current Ebola outbreak.

### Ethics Statement

Because the data were collected anonymously for the purposes of program evaluation (as part of the World Health Organization’s Ebola response activities) and the authors analyzed data that was de-identified and redacted, the study received an expedited review and exemption under the University of Florida Institutional Review Board (IRB) for the Protection of Human Subjects (IRB-02) #2014-U-1117. In accordance with the protocol detailed during a WHO research ethics review of the proposed data collection initiative, all research participants provided verbal informed consent and their names were not collected with the data. Participants were compensated for their time with US $5.00, and were informed that there would be no other direct benefit for participation in the study.

## Results

Community leaders shared with the research team their opinions regarding “best practices” concerning local community responses to the Ebola outbreak. Using a grounded theory approach, their feedback was analyzed for common themes, which generated an “ideal-type” [35] framework—a synthesis of commonly agreed-upon elements—for community-based response to the presence of Ebola in and near urban Liberian communities (Fig 1). This included three sequential phases of action and response: prevention, response and treatment, and sequelae. Both the findings and the figures represented in this section detail community responses, and suggest implications that concatenate with the existing literature on social structure, gender roles, healthcare capacity, and conflict histories in the region [34, 36–40].

### Prevention

Community leaders agreed that prevention was the best strategy to curtail the Ebola outbreak. During the Ebola outbreak in Monrovia, prevention was seen as including six key areas: (1) a sharp increase in the quantity and specificity of community-based training to prevent Ebola infection, (2) improved hygiene, sanitation, and the distribution of cleaning and protective materials, (3) creating a system of surveillance, (4) safely transporting infected individuals from the community into hospitals and Ebola Treatment Units (ETUs), (5) removal of the dead, and (6) establishing a community-based infrastructure to care for people who were sick with Ebola.

Training, said some, would counter the presence of fear in the communities and change the minds of those who continued to deny the existence of Ebola. Community leaders sought training to address the following key technical challenges in Ebola management:

- How to properly care for sick people
- How to isolate sick people
- How to manage quarantines safely
- How to administer community-based holding centers
- How to transport sick people safely
- How to isolate corpses
- How to bury infected corpses when corpse removal teams did not come
- How to maintain personal and household hygiene and use hygiene materials
- How to make use of available PPE
- How to properly disinfect their homes.

Notably, they were not seeking the basic information about Ebola then offered during health communications campaigns (ex. “What is Ebola? Have you ever heard of Ebola?”). Community leaders felt that they had a strong grasp on the basics of Ebola etiology and transmission, and the challenges they confronted or anticipated pertained to their uncertainty about how to respond in the context of health sector collapse. They called for training methods that would make public health messages more palatable and effective (by using local languages, video, door-to-door education, or billboards), but their concern was that people believed in Ebola and knew enough to be afraid, but not enough to respond effectively in the absence of a functioning Ebola medical response system or general healthcare sector. As one person reported, “We have heard the messages, but most people do not know how to practicalize them.” Continuity of messages and continuity of message delivery was highlighted as a critical issue. Community leaders emphasized the need to engage with communities on a daily basis, and for the messages they were relating to be correct and similar. Proposed training models included youth awareness workshops, community-based volunteer/training-of-trainer (ToT) workshops, and trainings for community leaders on the *management of* community-based Ebola responses.

To prevent the incidence of Ebola in their communities, community leaders argued that heightened attention to sanitation would prohibit the spread of the virus through bodily fluids. Governmental, NGO, and bilateral support was requested to sponsor heightened sanitation in private and public latrines, to include the distribution of buckets, ash, and bleach for washing, and locally obtainable personal protective equipment (PPE) gear like raincoats, rain boots, and plastic bags. As some demanded, “we need the same PPEs given to the medical doctors and nurses to be given to the community.”

Sanitation alone would not get the job done, however. In order to protect the community from the introduction of Ebola, and deter the spread of infection within neighborhoods, community leaders called for heightened surveillance efforts, and reported those in which they were already engaged. There was a strong community-based ethos informing control measures. As one community member said, “as a community we keep watch over each other.”

Community leaders’ accounts of optimal community-based Ebola surveillance included a four-tiered system of surveillance that was designed to prevent introduction of the virus into the community, facilitate reporting, disseminate information, sustain house-to-house monitoring, and support whistle blowing when community-members were non-compliant with EVD prevention protocols (Fig 2). The first level of surveillance involved the exclusion of “strangers” from the community, prohibiting visitors from sleeping in one’s home (for fear that they might be running from the presence of Ebola infection in their own home), and mandating a 21-day waiting period for those who wished to move into the community to insure that they were Ebola-free. The second level of surveillance included the formation of a community task force that would enforce the exclusion of strangers, and would also assume a leadership role in prevention (like keeping community members away from sick people or the dead). The community task force was also suggested to be responsible for alerting community members to the presence of Ebola, monitoring the health of the sick and their family members, engaging in reporting, and managing resource provisions for community-based quarantines and isolation. At a third level of surveillance was the block watch team. Community leaders suggested that block watches could go house-to-house to monitor the sick, refer new cases to health facilities, and identify efforts to conceal sickness or burials. Fourth, individuals within households were expected to invest in their own domestic surveillance for Ebola by reporting cases of illness within their household, removing themselves or their family members from the possibility of contagion upon discovering sick individuals, and even isolating themselves so as not to infect family members.

The division of labor suggested in community surveillance was implicitly-–and sometimes explicitly—gendered. Women and men were both included in community leadership focus groups, and their reports and ethnographic evidence suggests that men were expected to serve on community task force teams, block watch teams, or community action teams to keep strangers out and engage in reporting and whistle blowing. There was some concern about remilitarization, violence, and destabilization due to this trend, as was born out during the West Point riots in Monrovia, and in armed confrontations between male youth and police and Ebola response teams in Sierra Leone. The mobilization of young men in these communities can, and often does, involve a range of martial and surveillance-like behaviors that can turn rather quickly into a remilitarization of social organization [41–42].

As we will further elaborate below, women, on the other hand, were expected to engage in domestic surveillance, to monitor the physical wellness or illness of family members while they washed, clothed, and fed children, spouses, siblings, and elderly persons, and care for the sick. As a counterpoint, the domestication of surveillance among women caring for the bodies of others within households might have put women at a greater risk of infection within the household, especially under quarantine and isolation conditions, while men were more likely to be infected outside of the household (e.g. through transportation and porterage activities).

Most importantly, community leaders argued that substantial investments in local infrastructure and systems were required to prevent the spread of the epidemic, recalling Paul Farmer’s much circulated call for “staff, stuff, and systems” [9]. They requested government and other organizational support to create community “holding centers” to serve as interim sites for the sick and dead while waiting for Ebola response teams and/or burial teams to arrive. They demanded a hotline system that prioritized rapid response to local communities’ calls to place sick people in hospitals and ETUs and remove bodies. Community leaders also recommended a broader local communications infrastructure, including a better-staffed call center, more ambulances, the establishment of mobile clinics or the reopening of local community clinics that had closed their doors, more testing centers, and finally, the training of additional health workers and burial teams. These health workers, they insisted, need to be paid and given adequate benefits.

### Response and Treatment

Nearly all of the demands for the infrastructure improvements noted above derived from the experience of the failed international and national Ebola response observed and known to community leaders in the months of July, August, and September 2014. When community leaders called for the creation of a communications infrastructure, it was because their calls to hotlines had gone unanswered. When they called for the training of community members to provide care to the sick, manage holding centers, administer quarantines, and isolate or bury the dead, it was because they had experienced the social, medical, and ecological consequences of response teams *not* arriving in a timely manner or failing to arrive all.

Communication, or the lack thereof, constituted a critical part of the response’s failure at the juncture of prevention and response—the removal of the sick and the dead from communities. The anonymity involved in the process of removing the sick and the dead terrified people, and played a role in their decisions to avoid ETUs and hospitals and to conduct secret burials. In one case, a community leader reported losing an infected individual who had left the community for an “unknown destination.” After going from hospital to hospital, there was no record of that individual’s registration, death, or departure. This was seen as evidence of the failure of the international and national Ebola response.

In the absence of open clinics and hospitals, residents tried to assume responsibility for all aspects of healthcare in their local communities. Everything that follows in this section builds upon this premise. When community leaders engaged in discussions of “best practices” regarding response and treatment, community leaders agreed that the true “best response” was to obtain care in a hospital or ETU, to seek the removal of sick individuals by healthcare teams working for the government, and to engage in proper burials that reduced disease transmission. But, if resources were not in place, community leaders engaged in creative planning and response by innovating alternatives to how a community might best manage the presence of Ebola infection and dispose of infectious corpses.

This section details how community members prioritized the triage and treatment/care of unknown sickness and unexplained death in their communities. In order to assess how community members were likely to respond to illness in local communities, researchers asked a series of questions pertaining to their identification and management of illness in family, friends, and neighbors (Fig 3). Community leaders were quick to highlight the unnecessary morbidity and mortality due to preventable and treatable diseases, injuries, and basic health problems that were caused by the widespread closures of medical clinics and hospitals [43]. Most community leaders concurred that community members would be impelled to provide the best care they could offer for their families within their homes. Demands for guidance abounded: “We need to know how to protect ourselves while taking care of the sick.” “We want training and materials for how we can handle ourselves and the dead.” In order to align local realities with public health messages that were becoming increasingly unreasonable due to their disconnect with the lack of available services, they asked that community taskforce members be trained in basic healthcare provision, treatment of symptoms, and corpse removal.

When someone within a household fell ill, community leaders reported that they were first cared for within the home with palliative care. The person was administered “first-aid treatment,” including locally available pharmaceuticals, herbal remedies, locally accessible oral rehydration solutions or therapies, the provision of fluids, and the early administration of anti-malarials. It was not expected that any single household would be able to provide all of these interventions.

If the sick individual did not recover, community leaders expected caregivers to take a sick person to a hospital, or call the health team. There was a general consensus that if a person did fall ill, both health teams and the community must be notified so that they could respond and take precautions. However, the technical details regarding the proper handling of sick community members at home and in transit was ambiguous among respondents because it was ambivalent in the community. Transportation itself was a vector for infection. Leaders recounted examples of people who had walked to the hospital or carried sick children in their arms rather than travel by taxi or ask for help with transportation, in order to prevent the spread of infection.

Caregiving in all aspects demanded physical contact, but the public health messages regarding physical contact failed to recognize this reality. As some respondents noted, one message said, “Don’t touch,” while another said, “Touch, but use plastic gloves.” The lack of consistency may have resulted in experimentation and innovation, but it also elevated local perceptions that the message “Don’t Touch” was impractical and unhelpful in the context of immediate need. This was particularly significant in a social milieu in which nearly every able-bodied individual functioned as at least a part-time caretaker for children, elderly, or physically or mentally disabled friends and relatives.

Instead, community-based quarantine was identified as the best available strategy or approach. The process of quarantine required careful oversight and supply of resources, and community-leaders gave careful thought to how they might best support individuals and families in isolation and quarantine. In community leaders’ discussions, it was apparent that they sought to position the community at the center of the Ebola treatment response by managing the health and safety of quarantined families through food supply, illness surveillance and oversight, reporting, the provision of medical supplies, and communication and information. There was a strong willingness on the part of the community to serve as a central axis for interaction between the state and local individuals and families by doing the work of organizing food, medical, hygiene, and PPE distribution, case identification and surveillance, multi-level communication and reporting, and patient and corpse conveyance.

Community-based quarantine and home-based healthcare, however, caught communities in a Catch-22. Many community leaders were afraid of continued epidemic spread in their communities, and believed that health workers’ training, medicine, and materials prepared them to support and treat the sick [44]. Conversely, as one research team member commented in her field notes from one focus group, if a relative or neighbor was ill, just half of respondents were likely to call a health team hotline or encourage that person to go to a hospital or Ebola Treatment Unit (ETU). They had good reasons not to. By the time of this study, many of those who had gone to hospitals and ETUs had never returned, or had been turned away from multiple facilities due to lack of beds. Some were concerned that the failure to report the deaths of Ebola patients to their families and communities meant that loved ones’ bodies had been dissected for body parts after death, and that foreign NGOs were trying to keep the practice a secret [44].

This research offered direct insight into the fore-planning process of women as they consider how to respond if and when Ebola arrives in their households, families, and social networks. A broad subset of respondents—mainly women—reported that they would care for their sick family members on their own, and that they preferred to do so inside the home. They described a plan for isolating themselves with a sick family member[s] and for providing the best locally available appropriate care they could offer, using available resources (Fig 4). As one woman noted, “It will be impossible that my child or husband is sick and I refuse to touch them. I do not have the courage or heart to do that.” Referencing a widely circulated video of a nurse who had made her own personal protective equipment (PPE) from garbage bags, rain coats and boots, and gloves, an elderly woman reported her intention of making her own PPE from locally available materials. “I will find my own PPE (using a raincoat, plastic bags on hands) and care for sick relatives like I saw on television. If the person is not getting better, I will hold them (with the plastic still on my hands) and take them to the hospital.” Women showed an intense conviction that they should care for their families, and showed a desire to do so, despite risks to their own health.

Childcare, eldercare, home-based healthcare and the graduated triage approach were gendered activities; and home-based care constituted a zone of risk for both predominantly female caregivers and their dependents [45], in contrast to community-based surveillance, education, and transport roles, which functioned as a zone of risk for men. This may have also had unrecognized repercussions on infant and child mortality, and on the now-confirmed explosive chains of Ebola transmission within kinship networks and families during the Ebola crisis [46].

Community leaders reported that, after parents infected with Ebola had been removed to hospitals or had died, their children were placed under community quarantine for 21 days. During this time:

“Many of these children do not survive their quarantine periods; they just cry to death because no one can provide care from the outset. Community members are unable to help because of fear of the Ebola virus. Babies and young children are dying, not from Ebola, but because there is no one to care for them and the health workers’ response to these children is too slow.”

The reported deaths of young children under quarantine paint a challenging picture to communities’ descriptions of providing care (food and water) to local families and children throughout the quarantine period. It also facilitated the impression that women’s greater involvement in the direct support of children’s diet and healthcare and in traditional burial practices was resulting in a gender differential in levels of exposure, although this has not been borne out by the official data [47–48, 22]. A community-based approach lends itself to the interpretation that, although male and female case incidences and fatalities were roughly equal [2], women’s risk of mortality may have been impacted by their greater reluctance to seek early treatment for Ebola out of fear for children’s lives, and due to concern for children’s and dependents’ well-being under the quarantine that would follow their hospitalization at an Ebola treatment unit (ETU) or temporary quarantine at community-care centers (CCCs).

Other responses indicated the existence of a path for Ebola response in the community that diverged from both formal healthcare seeking and home-based care. The retreat and isolation of sick individuals was characterized as a protective measure for the community, and as an act of generosity from sick individuals to the community. Survivors characterized their own self-isolation as a strategy for honoring and caring for the families of people who had died. Unsupported quarantine entailed the isolation of an infected individual, without providing treatment to him/her, or referring him/her to a healthcare facility. As one person noted, “If my son is sick, I will run away from him. I am not a health worker to tell whether it is Ebola or malaria. It will be better he dies alone and I be left behind to care for his sisters and brothers.” The intention of this statement sounds chilling, but its meaning is complex, and considers the futures of the people surrounding the individual who present as terminally ill, with little likelihood of treatment or survival.

### Sequelae

The legacies of Ebola in Liberia appear to be drastic and long-lasting [48]. This study identified three critical issues pertaining to the sequelae of Ebola: (1) the reintegration of Ebola survivors into local communities, (2) the care and management of “Ebola orphans”—or children who had lost one or both parents to Ebola, and (3) memorialization of individuals who have died of Ebola.

At the height of the outbreak, when these data were collected, focus group participants reported support and acceptance for Ebola survivors in the community. Ebola survivors were widely recognized as being an asset in the fight against Ebola. They were seen as being an embodiment of positive messages suggesting that early treatment could allow one to survive Ebola, and they were referred to many times as “ambassadors” of Ebola awareness, as “living testimony to the Ebola crisis,” and as positive role models. Within focus groups, survivors already appeared to be accepting their positive role model status, and offered vignettes like the following:

1. I am a native doctor (herbalist). I used to heal sick people before I got sick with Ebola. After I cured a few people, there was one person who I was asked to treat. Before I got there the person had already died. I didn’t touch the dead body. When I started feeling sick and noticed that I was showing signs of Ebola I walked to the hospital all by myself. I care for my family and didn’t want to get them infected. I was also afraid to get other people infected, so I didn’t ride a taxi or motorbike; I walked to the ETU. From the beginning, I was discouraged, I lost hope because people who were in the same room (on different beds) died. I spent six days and started recovering. I was given material to use (gloves, PPE, chlorine) and asked to help anyone that I saw showing the signs of Ebola and encouraged people to go to the ETU early.
2. It was traumatic for me but I thank the Almighty God and the medical staff at ELWA for surviving this outbreak. For me when I started feeling sick, I went to the treatment center right away to seek early treatment. I think that is how I survived. Most of the people who come to the center do not come early to get help, and I think that is why people are dying. My advice to people is that they should seek early treatment when they start to feel sick and see signs and symptoms of Ebola.

Although some community leaders mentioned that they were afraid of survivors, most indicated that they welcomed the return of Ebola survivors into their community. They acknowledged that they understood that they were no longer infectious, and that these individuals could not be re-infected with the virus. Concern was voiced that the Ebola virus was found in semen for up to three months following infection, and that previously infected individuals must take care to avoid sexual relations. Others conflated this three-month time period with the perception that these individuals were still infectious, and recommended that they be placed in a halfway house for three months following an infection.

The care of children orphaned by Ebola was widely regarded as a communal responsibility. Some community members mentioned that children would be brought into their homes and families, noting, “The children become our children. These children are our own because their parents are no more.” Another commented that the community leaders must, “encourage people to take children whose parents have died of Ebola as their own, because we have lived in the same community for years and they are like family to us.” Recalling the war and the fragility of life in urban Monrovia [34], one individual noted, “We have done it before. We will take care of the children. Education, feeding, and shelter.” Several respondents mentioned that children required psychosocial counseling to recover from “traumatization” due to having lost their parents, and they recommended that NGOs make counseling available to Ebola orphans in communities and in orphanages. They also stipulated that orphans should not be forced to endure stigma or discrimination because their parents had died of Ebola.

Despite the will to care for the children, there was considerable concern about the economic, emotional, and residential burden that additional children would impose. When children had extended families elsewhere, communities felt that they should be united with them, and that NGOs must assist with “family reunification.” Others called for the creation of orphanages within their communities so that community members could oversee the development of the children, while delegating financial and educational costs to NGOs, UNICEF, the World Health Organization, and the MOHSW. The majority of individuals, however, seemed to hope that children would be able to stay within the community, but would receive financial support for their clothing, food, and education from governmental, non-governmental, ecumenical, and charitable sources.

The data from this research indicated the need for some form of widespread and public memorialization of the lives lost to Ebola. Community members called for: a National Memorial Day; the construction of a statue in the middle of the community; a formal memorial service at the end of the outbreak; a parade; or a day called “Black Day” to be recognized by law. Community members also recommended that a mass grave be built with a headstone inscribing the names of all who had died of Ebola, and had been cremated. The goal of this memorial is to provide family members with a space to “remember” and pay tribute to their loved ones by visiting the grave and laying flowers. Additional suggestions were practical, and included providing scholarships, financial aid, general support, and counseling to Ebola orphans.

## Discussion

While the research reported here takes considerable strides towards helping to understand how local communities in Liberia responded, and envisioned their response, to Ebola, this information must not be mistaken as an indication of community political, medical, or social empowerment or institution-building—although this, too, was present. These communities were not empowered, they were desperate and often abandoned. They found resources from within their communities to compensate for the collective failure of state and international institutions to implement systems of surveillance, treatment, and response. What we are observing here is a community-based response to a condition of medical statelessness and structural violence [49–50].

Even so, health sensitization efforts continued to emphasize the ‘low-hanging fruit’ of public health communications [51] throughout the response, repeating messages like: “What is Ebola? How is it spread? What are the symptoms? How long does it last?” But community health messaging essentially failed to provide the kinds of ‘higher-order,’ practical information and training that communities were desperate for, like “How do I manage a family of children, including infants and toddlers, in quarantine?” “How do I transport someone to a hospital or clinic without promoting infection?” “What capacities need to be built to support a holding center?” “What does my community do with an exposed and infectious body when the health teams do not come to collect it?” “What can I do to make sure that you don’t lose or steal my father/brother/sister/mother at your health facility?”

In the future, engaging local communities in epidemic response will require answering their challenging questions about their encounters with systemic failures *in real time*. Communities sought guidance for triaging a sick person when he or she had been turned away from hospitals, for building and supporting holding units in communities, and for reporting deaths when their calls to hotlines went unanswered. The global health community needs to consider what it would mean to put into place surveillance and reporting mechanisms in which community-based leaders have the ability to directly account for health, illness, or death of every individual in the population. This could be done through the creation of health identification numbers, the creation of health census lists, or other mechanisms of reporting and marking. In a context in which every death is an Ebola death because there are no community-based testing facilities for Ebola, every death needs to be counted as worthy of being reported. (And when everyone has a number, everyone counts.)

But can locally affected populations, in effect, govern themselves by engaging in medical self-surveillance, self-management, and self-triage? The ethnographic evidence suggests that they can indeed do so [22,52–53], whilst the public health literature has previously examined the importance of local surveillance in other hemorrhagic fever outbreaks in Africa [54–55]. In the short-term, risk can be moderated, in part, by ensuring that required daily resources like food, medicine, housing, PPEs, and other resources are in abundance, and that informational demands meet local populations “where they are.” In the long term, efforts need to focus on equipping local communities with the material and knowledge resources to respond to Ebola and to help build a surveillance infrastructure that can inform a stronger post-epidemic local and state governance architecture.

The study also suggests that the gendered distribution of morbidity and mortality in this Ebola outbreak is strongly associated with existing relations of caregiving and with the distribution of labor in community surveillance and response. The most important thing to understand about culture and caregiving is that women are not going to abdicate the role of primary caregivers. Indeed, the data collection exercise offered direct insight into the fore-planning process of women as they consider how to respond if and when Ebola were to arrive in their households, families, and social networks. Resources must be set into motion to support men and women in their community-allocated surveillance roles and to support women in their caregiving roles, in order to engender support for local-international collaboration and connect the Ebola response effort to the lived experiences of local persons.

### Limitations

This study had two major limitations. First, the number of participants in the focus groups was large (15–20). In order to address this issue, the PI stationed four research assistants around the group in order to capture the responses of all participants. Second, the data collected were partly conjectural, and questions were posed as hypothetical or focused on best practices, rather than on direct local experiences and actions, in order to avoid issues of concealment or avoidance during data collection. As a result, community leaders’ feedback is regarded by the researchers as an “ideal-typical” representation of what a community-based response to Ebola should have been like, rather than a factual account of how these same communities actually responded to the incidence of Ebola. Respondents shared information that was based on their knowledge of Ebola, on community and government messages that they had received about Ebola, on resources that they sought for their communities, and on their experiences with Ebola and non-related Ebola morbidity and mortality.
