# Correction: Community-Centered Responses to Ebola in Urban Liberia: The View from Below

**DOI:** 10.1371/journal.pntd.0003767

**Published:** 2015-05-07

**Authors:** Sharon Alane Abramowitz, Kristen E. McLean, Sarah Lindley McKune, Kevin Louis Bardosh, Mosoka Fallah, Josephine Monger, Kodjo Tehoungue, Patricia A. Omidian

There is an error in the affiliation for authors Mosoka Fallah, Josephine Monger, and Kodjo Tehoungue. The correct affiliation is: the World Health Organization.
